# Supplementary material for: Disease–Gene Networks of Skin Pigmentation Disorders and Reconstruction of Protein–Protein Interaction Networks
Source: Bioengineering (Basel). 2022 Dec 21;10(1):13. doi: 10.3390/bioengineering10010013 (PMC9854651; doi:10.3390/bioengineering10010013)
Supplement: Supplementary file 1 [file bioengineering-10-00013-s001.zip › Supplementary Figures DArcy et al FINAL.pdf]

Figure 1

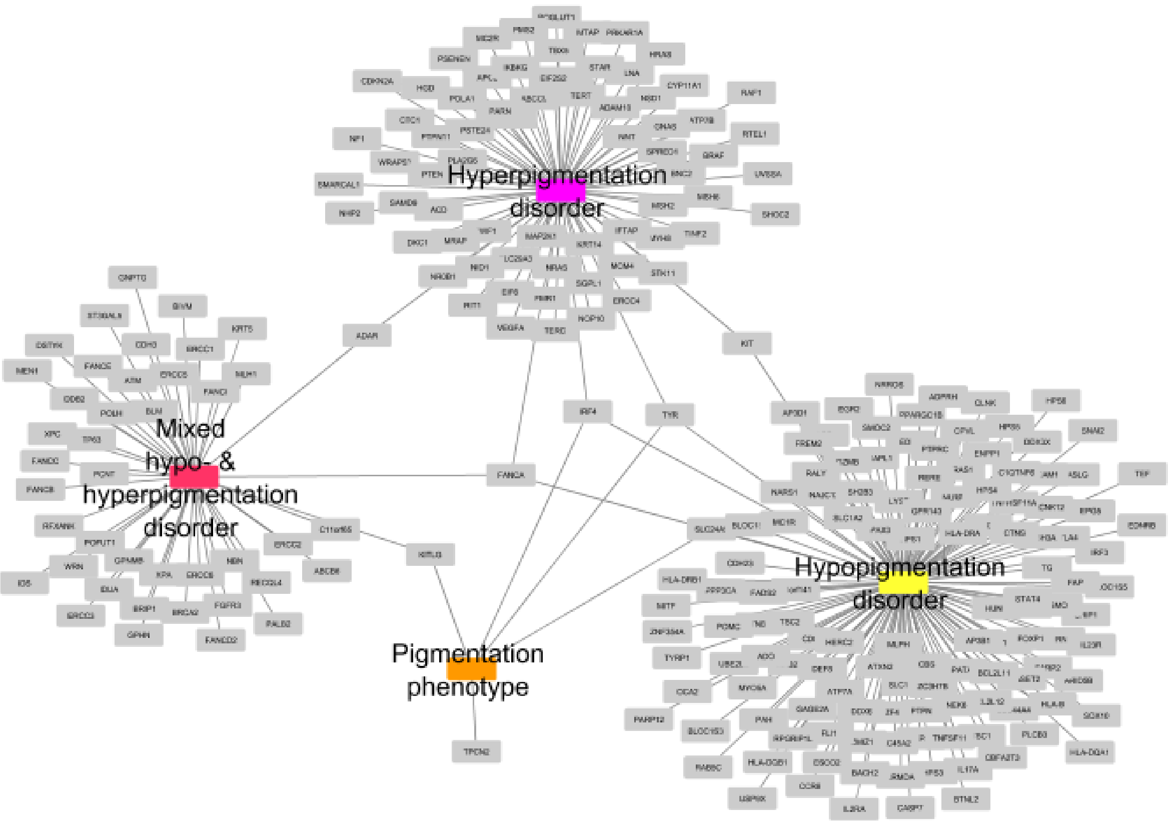

Figure S1. Disease-gene network of skin pigmentation disorders combined into subgroups. Genes are represented as rectangles in grey. Diseases are shown in triangles and coloured according to subgroup: hyper-pigmentation (pink), hypopigmentation (yellow), mixed pigmentation (red), and pigmentation phenotype (orange). The network was represented using Cytoscape.

(a)

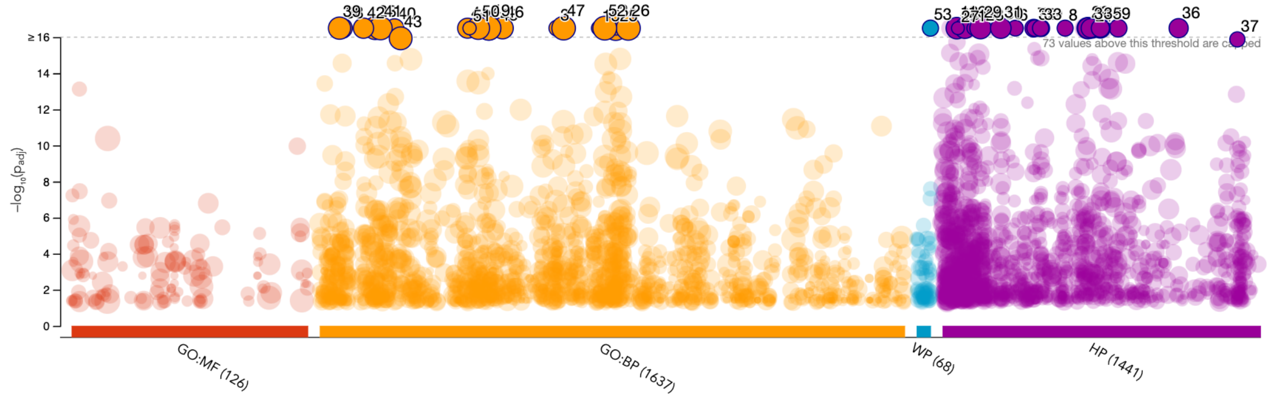

(b)

ID	Source	Term ID	Term Name	p_{adj} (query_1) ↑
1	HP	HP:0001000	Abnormality of skin pigmentation	4.842×10^{-95}
2	HP	HP:0001010	Hypopigmentation of the skin	1.996×10^{-76}
4	HP	HP:0000953	Hyperpigmentation of the skin	7.403×10^{-61}
7	HP	HP:0007400	Irregular hyperpigmentation	1.139×10^{-45}
8	HP	HP:0009887	Abnormality of hair pigmentation	1.227×10^{-45}
9	HP	HP:0012733	Macule	1.227×10^{-45}
3	GO:BP	GO:0043473	pigmentation	9.234×10^{-45}
6	HP	HP:0005599	Hypopigmentation of hair	3.535×10^{-44}
5	HP	HP:0001034	Hypermelanotic macule	5.340×10^{-44}
10	HP	HP:0001053	Hypopigmented skin patches	1.198×10^{-37}
11	HP	HP:0001480	Freckling	1.619×10^{-37}
13	HP	HP:0011121	Abnormality of skin morphology	2.707×10^{-36}
14	HP	HP:0011355	Localized skin lesion	7.797×10^{-35}
12	HP	HP:0000951	Abnormality of the skin	5.214×10^{-34}
15	GO:BP	GO:0048066	developmental pigmentation	1.191×10^{-29}
20	HP	HP:0011138	Abnormality of skin adnexa morphology	1.012×10^{-27}
17	HP	HP:0002664	Neoplasm	4.771×10^{-27}
22	HP	HP:0011793	Neoplasm by anatomical site	6.003×10^{-27}
16	HP	HP:0001595	Abnormal hair morphology	1.338×10^{-26}
18	HP	HP:0002216	Premature graying of hair	6.306×10^{-26}
19	HP	HP:0008069	Neoplasm of the skin	6.306×10^{-26}
21	HP	HP:0011358	Generalized hypopigmentation of hair	6.306×10^{-26}
23	GO:BP	GO:0006950	response to stress	1.296×10^{-24}
24	GO:BP	GO:0050896	response to stimulus	2.191×10^{-23}
25	GO:BP	GO:0050931	pigment cell differentiation	2.191×10^{-23}
27	HP	HP:0001022	Albinism	1.319×10^{-22}
32	HP	HP:0007495	Prematurely aged appearance	1.319×10^{-22}
38	GO:BP	GO:0002520	immune system development	1.554×10^{-21}
42	GO:BP	GO:0006259	DNA metabolic process	5.014×10^{-21}
26	GO:BP	GO:0051716	cellular response to stimulus	7.415×10^{-21}
51	GO:BP	GO:0030318	melanocyte differentiation	1.084×10^{-20}
35	HP	HP:0011792	Neoplasm by histology	1.928×10^{-20}
34	HP	HP:0011276	Vascular skin abnormality	3.169×10^{-20}
29	HP	HP:0002715	Abnormality of the immune system	4.388×10^{-20}
41	GO:BP	GO:0007275	multicellular organism development	6.792×10^{-20}
46	GO:BP	GO:0033554	cellular response to stress	7.923×10^{-20}
31	HP	HP:0004328	Abnormal anterior eye segment morphology	9.126×10^{-20}
30	HP	HP:0004377	Hematological neoplasm	1.528×10^{-19}
28	HP	HP:0002861	Melanoma	1.698×10^{-19}
33	HP	HP:0008034	Abnormal iris pigmentation	3.574×10^{-19}
49	GO:BP	GO:0032501	multicellular organismal process	3.977×10^{-19}
36	HP	HP:0032251	Abnormal immune system morphology	1.615×10^{-18}
48	GO:BP	GO:0032502	developmental process	1.875×10^{-18}
45	GO:BP	GO:0033059	cellular pigmentation	2.051×10^{-18}
50	GO:BP	GO:0031325	positive regulation of cellular metabolic process	1.359×10^{-17}
39	GO:BP	GO:0002376	immune system process	4.124×10^{-17}
52	GO:BP	GO:0048518	positive regulation of biological process	4.351×10^{-17}
47	GO:BP	GO:0044260	cellular macromolecule metabolic process	4.391×10^{-17}
44	GO:BP	GO:0030097	hemopoiesis	7.451×10^{-17}
53	WP	WP:WP4946	DNA repair pathways, full network	7.548×10^{-17}
40	GO:BP	GO:0009314	response to radiation	9.630×10^{-17}
43	GO:BP	GO:0009893	positive regulation of metabolic process	1.159×10^{-16}
37	HP	HP:0100585	Telangiectasia of the skin	1.328×10^{-16}

Figure S2. Functional analysis of all disease genes using g:GOSt gene set enrichment. (a) Manhattan plot rep-resenting the molecular function (MF), the biological process (BP), wikipathways (WP) and hu-man phenotype (HP). The y-axis is a –log(10) of the adjusted p-value, which is capped in this plot. (b). Table of the GO terms of the selected nodes in the Manhattan plot, ranked from high (purple) to low (green) using the adjusted p-value.

(a)

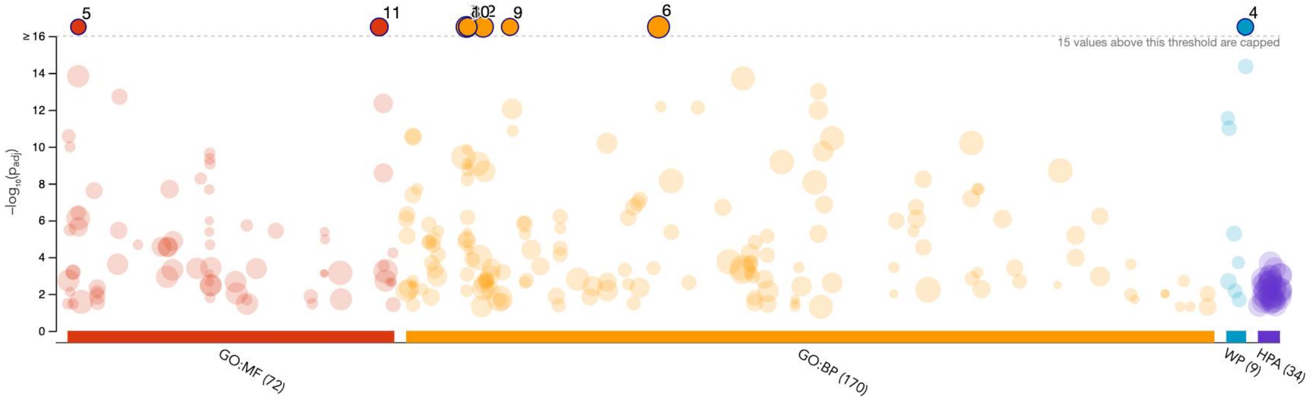

(b)

ID	Source	Term ID	Term Name	p_{adj} (query_1) ↑
1	GO:BP	GO:0006281	DNA repair	1.104×10^{-45}
4	WP	WP:WP4946	DNA repair pathways, full network	1.501×10^{-40}
2	GO:BP	GO:0006974	cellular response to DNA damage stimulus	6.029×10^{-40}
3	GO:BP	GO:0006259	DNA metabolic process	4.094×10^{-37}
6	GO:BP	GO:0033554	cellular response to stress	3.900×10^{-29}
5	GO:MF	GO:0003684	damaged DNA binding	1.058×10^{-27}
7	GO:BP	GO:0006289	nucleotide-excision repair	2.969×10^{-23}
9	GO:BP	GO:0009411	response to UV	5.585×10^{-22}
8	GO:BP	GO:0006302	double-strand break repair	8.298×10^{-22}
10	GO:BP	GO:0006310	DNA recombination	1.368×10^{-20}
11	GO:MF	GO:0140097	catalytic activity, acting on DNA	1.995×10^{-19}

Figure S3. Functional analysis of cluster 1 disease genes using g:GOSt gene set enrichment. (a) Manhattan plot representing the molecular function (MF), the biological process (BP), wikipathways (WP) and hu-man phenotype (HP). The y-axis is a –log(10) of the adjusted p-value, which is capped in this plot. (b). Table of the GO terms of the selected nodes in the Manhattan plot, ranked from high (purple) to low (green) using the adjusted p-value.

(a)

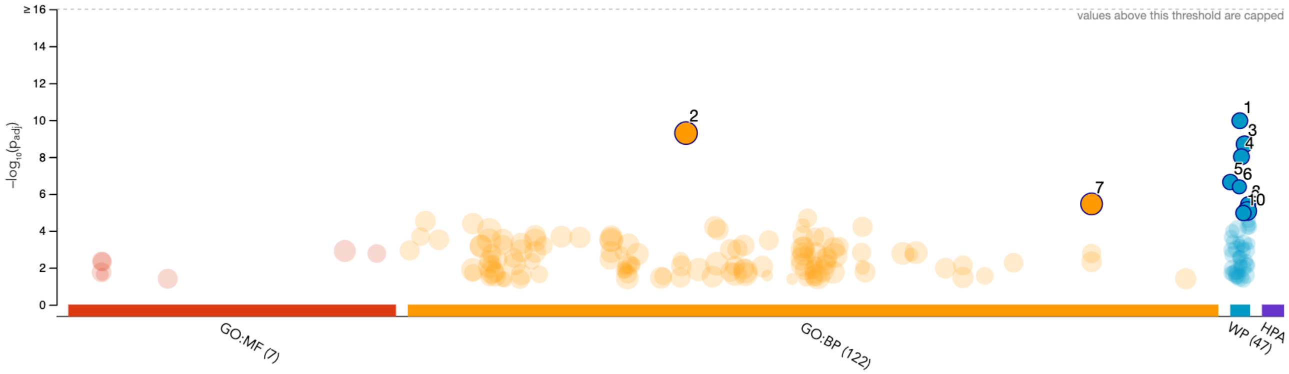

(b)

ID	Source	Term ID	Term Name	p_{adj} (query_1) ↑
1	WP	WP:WP2261	Glioblastoma signaling pathways	1.100×10^{-10}
2	GO:BP	GO:0035556	intracellular signal transduction	5.135×10^{-10}
3	WP	WP:WP4549	Fragile X syndrome	2.043×10^{-9}
4	WP	WP:WP4806	EGFR tyrosine kinase inhibitor resistance	9.796×10^{-9}
5	WP	WP:WP4685	Melanoma	2.314×10^{-7}
6	WP	WP:WP3676	BDNF-TrkB signaling	4.182×10^{-7}
7	GO:BP	GO:1902531	regulation of intracellular signal transduction	3.531×10^{-6}
8	WP	WP:WP4539	Synaptic signaling pathways associated with a...	3.662×10^{-6}
9	WP	WP:WP4172	PI3K-Akt signaling pathway	8.561×10^{-6}
10	WP	WP:WP4155	Endometrial cancer	1.103×10^{-5}

Figure S4. Functional analysis of cluster 2 disease genes using g:GOSt gene set enrichment. (a) Manhattan plot representing the molecular function (MF), the biological process (BP), wikipathways (WP) and hu-man phenotype (HP). The y-axis is a –log(10) of the adjusted p-value, which is capped in this plot. (b). Table of the GO terms of the selected nodes in the Manhattan plot, ranked from high (purple) to low (green) using the adjusted p-value.

(a)

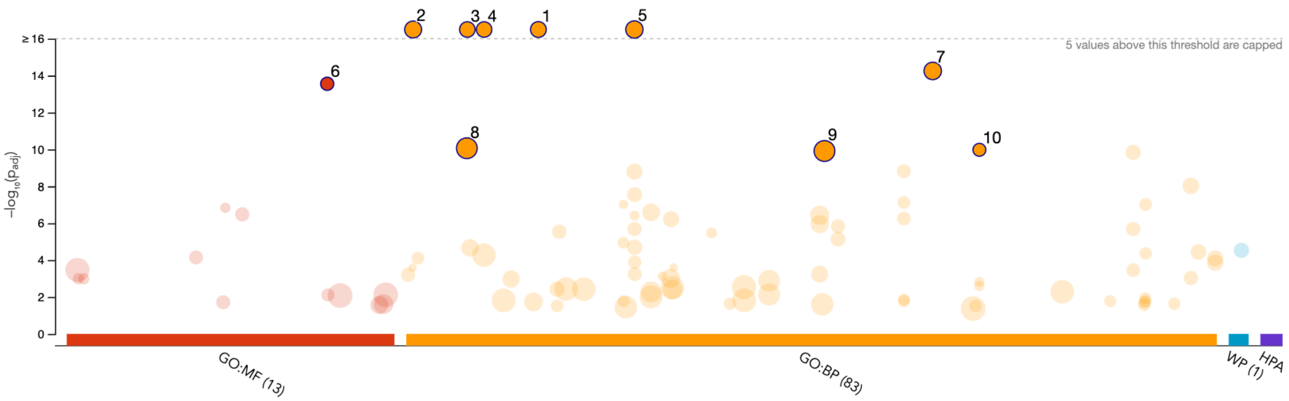

(b)

ID	Source	Term ID	Term Name	p_{adj} (query_1)
1	GO:BP	GO:0010833	telomere maintenance via telomere lengthening	7.175×10^{-22}
2	GO:BP	GO:0000723	telomere maintenance	2.646×10^{-19}
4	GO:BP	GO:0007004	telomere maintenance via telomerase	4.071×10^{-19}
3	GO:BP	GO:0006278	RNA-templated DNA biosynthetic process	4.653×10^{-19}
5	GO:BP	GO:0032200	telomere organization	1.872×10^{-18}
7	GO:BP	GO:0071897	DNA biosynthetic process	5.563×10^{-15}
6	GO:MF	GO:0070034	telomerase RNA binding	2.767×10^{-14}
8	GO:BP	GO:0006259	DNA metabolic process	8.602×10^{-11}
10	GO:BP	GO:0090670	RNA localization to Cajal body	1.049×10^{-10}
9	GO:BP	GO:0051276	chromosome organization	1.217×10^{-10}

Figure S5. Functional analysis of cluster 3 disease genes using g:GOSt gene set enrichment. (a) Manhattan plot representing the molecular function (MF), the biological process (BP), wikipathways (WP) and hu-man phenotype (HP). The y-axis is a –log(10) of the adjusted p-value, which is capped in this plot. (b). Table of the GO terms of the selected nodes in the Manhattan plot, ranked from high (purple) to low (green) using the adjusted p-value.

(a)

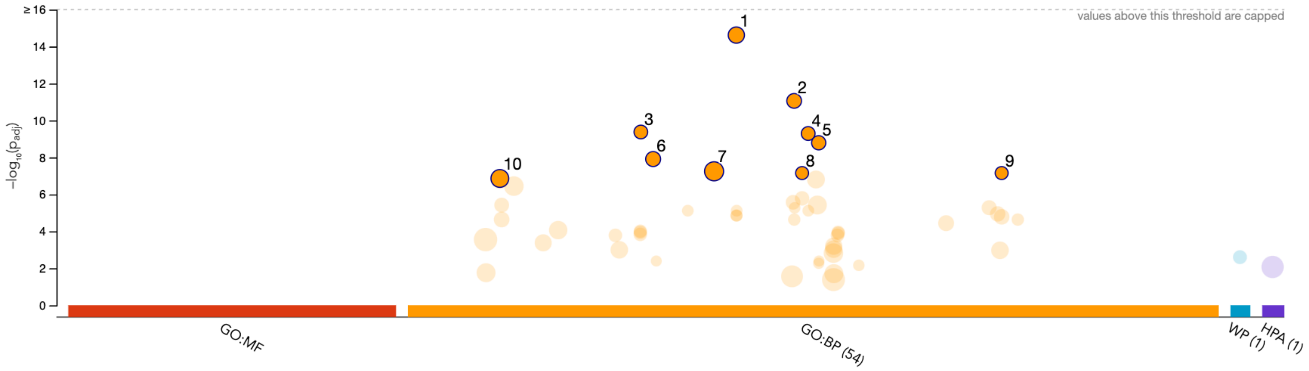

(b)

ID	Source	Term ID	Term Name	p_{adj} (query_1) ↑
1	GO:BP	GO:0043473	pigmentation	2.405×10^{-15}
2	GO:BP	GO:0048066	developmental pigmentation	8.723×10^{-12}
3	GO:BP	GO:0032438	melanosome organization	4.216×10^{-10}
4	GO:BP	GO:0048753	pigment granule organization	5.094×10^{-10}
5	GO:BP	GO:0050931	pigment cell differentiation	1.615×10^{-9}
6	GO:BP	GO:0033059	cellular pigmentation	1.226×10^{-8}
7	GO:BP	GO:0042060	wound healing	5.662×10^{-8}
8	GO:BP	GO:0048490	anterograde synaptic vesicle transport	7.022×10^{-8}
9	GO:BP	GO:0099517	synaptic vesicle transport along microtubule	7.022×10^{-8}
10	GO:BP	GO:0007596	blood coagulation	1.376×10^{-7}

Figure S6. Functional analysis of cluster 4 disease genes using g:GOSt gene set enrichment. (a) Manhattan plot representing the molecular function (MF), the biological process (BP), wikipathways (WP) and hu-man phenotype (HP). The y-axis is a –log(10) of the adjusted p-value, which is capped in this plot. (b). Table of the GO terms of the selected nodes in the Manhattan plot, ranked from high (purple) to low (green) using the adjusted p-value.

(a)

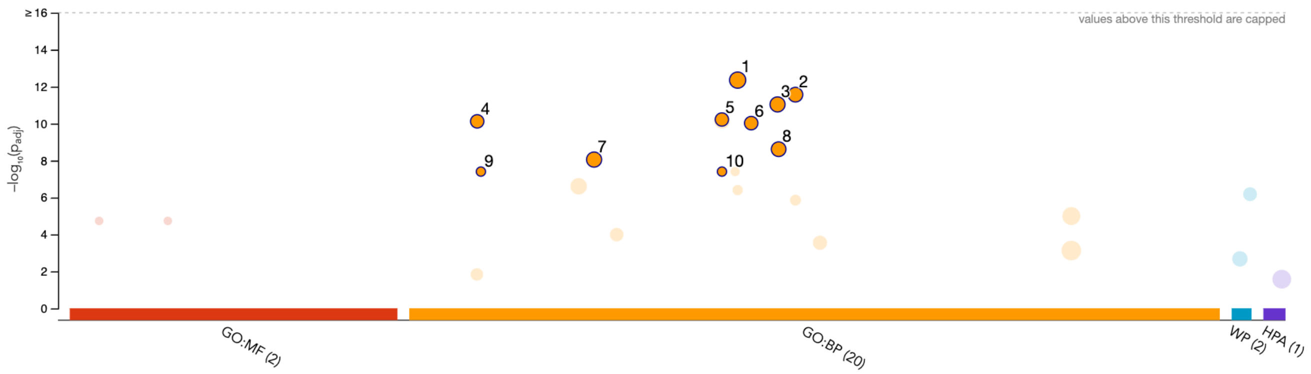

(b)

ID	Source	Term ID	Term Name	p_{adj} (query_1) ↑
1	GO:BP	GO:0043473	pigmentation	4.430×10^{-13}
2	GO:BP	GO:0048066	developmental pigmentation	2.718×10^{-12}
3	GO:BP	GO:0046148	pigment biosynthetic process	9.180×10^{-12}
5	GO:BP	GO:0042438	melanin biosynthetic process	5.997×10^{-11}
4	GO:BP	GO:0006582	melanin metabolic process	7.575×10^{-11}
6	GO:BP	GO:0044550	secondary metabolite biosynthetic process	9.467×10^{-11}
8	GO:BP	GO:0046189	phenol-containing compound biosynthetic pro...	2.437×10^{-9}
7	GO:BP	GO:0019748	secondary metabolic process	8.885×10^{-9}
9	GO:BP	GO:0006726	eye pigment biosynthetic process	3.975×10^{-8}
10	GO:BP	GO:0042441	eye pigment metabolic process	3.975×10^{-8}

Figure S7. Functional analysis of cluster 5 disease genes using g:GOSt gene set enrichment. (a) Manhattan plot representing the molecular function (MF), the biological process (BP), wikipathways (WP) and hu-man phenotype (HP). The y-axis is a –log(10) of the adjusted p-value, which is capped in this plot. (b). Table of the GO terms of the selected nodes in the Manhattan plot, ranked from high (purple) to low (green) using the adjusted p-value.

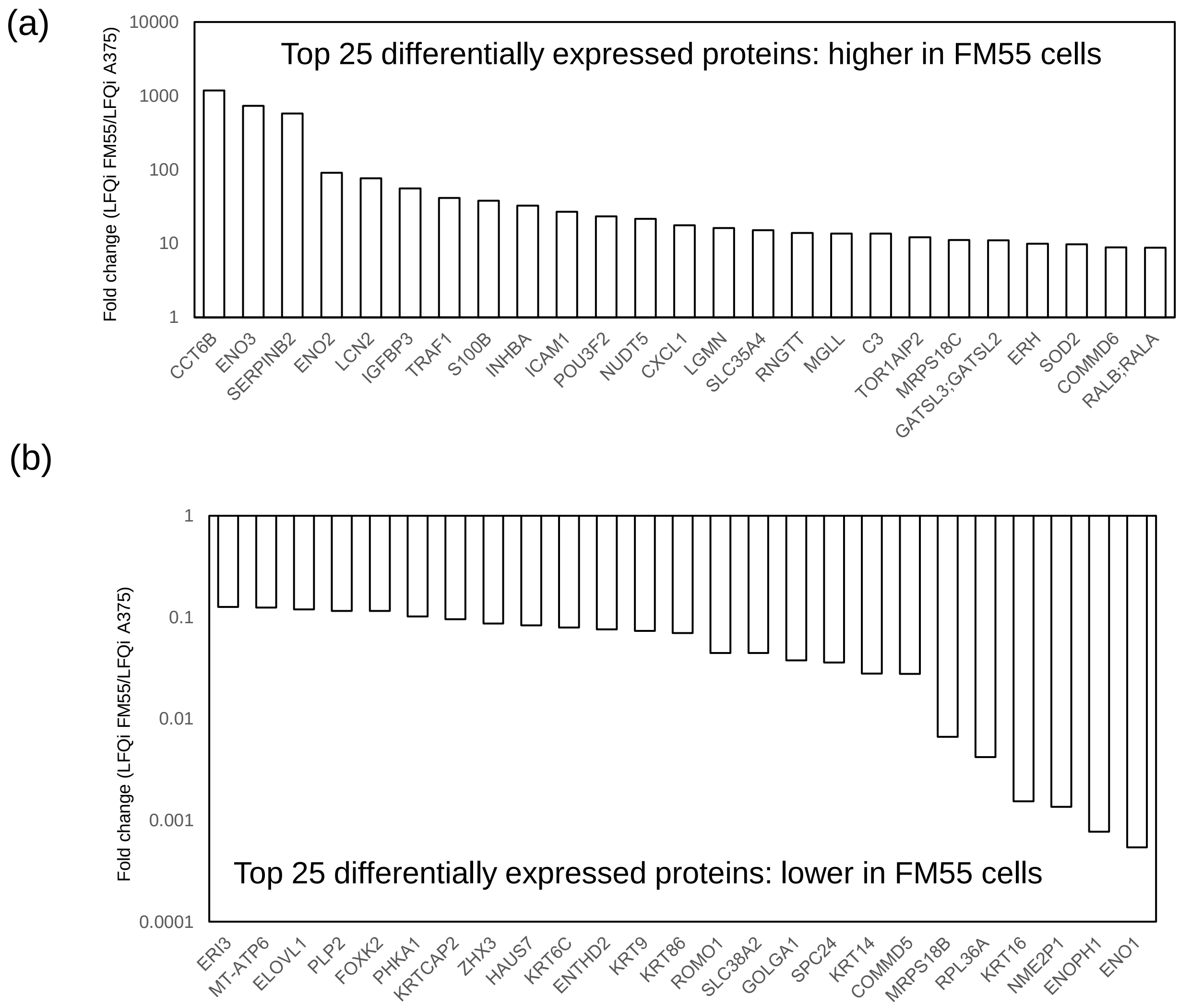

Figure S8. Top 50 differentially expressed proteins comparing A375 and FM55 cells. (**a**) Top 25 proteins differentially higher expressed in FM55 cells. (**a**) Top 25 proteins differentially lower expressed in FM55 cells.
